# The impact of colonial and contemporary land policies on climate change adaptation in Zimbabwe’s communal areas

**DOI:** 10.4102/jamba.v14i1.1311

**Published:** 2022-10-28

**Authors:** Trymore Maganga, Catherine Conrad Suso

**Affiliations:** 1 Department of International Development Studies, Saint Mary’s University, Halifax, Canada; 2 Department of Geography and Environmental Studies, Saint Mary’s University, Halifax, Canada

**Keywords:** colonial policies, climate change, food security, small-scale farmers, adaptation, livelihoods

## Abstract

The main aim of this article was to examine the impact of colonial and contemporary development policies on climate change adaptation among communal farmers in Zimbabwe. As such, the objective was to document and better understand how the legacy of colonialism, coupled with the current climate change impacts is making adaptation a serious challenge for farmers in particular regions of the country. This study was conducted in Zimbabwe’s Buhera Rural District (Ward 30) and Chipinge Rural District (Ward 11). Data collection involved the use of individual household interviews, with the use of a snowball sampling method, focus group discussions, key informant interviews and direct observation in the field. It was found that the lack of income diversity opportunities has further exposed several livelihoods to climate change and compromised their abilities to respond and recover under periods of climatic stress. It was ascertained that the adaptation challenges experienced by African farmers were brought about by the colonial land system that evicted them from their customary lands and allocated them land in poor agroecological regions that fail to support production. The authors argue that climate change adaptation challenges in communal areas should be understood from a colonial and historical development perspective that led to the establishment of communal farming zones. There is also a need to understand climate vulnerability in the context of post-independence development strategies that have led to the underdevelopment of peasant agriculture and reduced farmers’ ability to adapt to climate change.

**Contribution:**

Climate change adaptation policies should recognise the country’s colonial and historical legacy that has led to poverty and other livelihood challenges in communal areas. By acknowledging this, policymakers are better positioned to understand the structural issues making adaptation difficult, and they could intervene by proposing context-specific adaptation strategies that meet the needs of communal farmers.

## Introduction

### Colonial land policies and communal livelihoods

Zimbabwe has a long history of suffering among small-scale farmers that can be traced back to the colonial period (1890–1980) when the country was under the rule of Europeans. During this period, the British Government, under the British South African Company (BSAC)^1^ that was fronted by Cecil John Rhodes, enacted several land concessions and treaties that included the Lippert Concession (1889); Native Reserves Order in Council (1898) and Land Apportionment Act (1930) to grab prime agricultural lands from native African farmers. Using the Lippert Concession, the Europeans obtained:

[*T*]he sole and exclusive right, power and privilege for the full term of 100 years’ layout, grant or lease, farms, township buildings, plots, and grazing areas; to impose and levy rents, licenses and taxes thereon and to get in; collect and receive the same for his benefit; to give and grant certificates for the occupation of any farms, township, building, plots and grazing areas. (Mafa et al. 2015:38)

This treaty marked the beginning of the suffering that African farmers had to endure at the hands of the Europeans. As the years progressed, the number of Europeans coming into Zimbabwe (then Rhodesia) grew year by year because of the favourable agricultural policies that were presented to them by the BSAC (eds. Crush & Tevera 2010:55).

**FIGURE 1:**
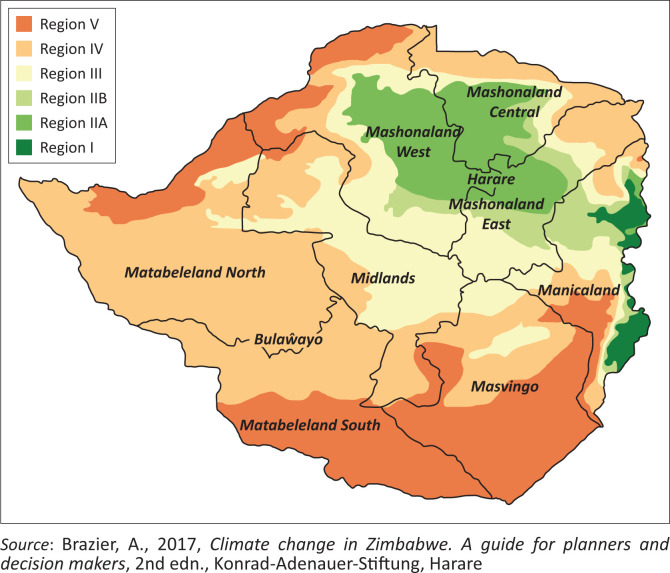
Zimbabwe’s agro-ecological regions.

In enticing more Europeans to settle and farm in the country, the BSAC sold land at cheaper prices and also introduced the contract labour system to provide white farmers with cheap labour to support their agricultural production (eds. Crush & Tevera 2010:56). These conditions that were brought about and promoted by the BSAC saw thousands of European farmers flocking into the country. This exacerbated the need for farming land for European farmers while encouraging evictions in African-owned farming lands (eds. Crush & Tevera 2010:56). The BSAC enacted several policies that include the Native Reserves Order in Council (1898) and the *Land Apportionment Act* (1930) to confiscate farming land in African reserves and redistribute it to the Europeans. Consequently, European farmers later settled in the country’s prime agricultural lands in agro-ecological regions (AER)^2^ I, II and III (see Figure 1), while African farmers were allocated land in poor AER IV and V or in ‘native reserves’ (Mafa et al. 2015:40; Potts 2010:79). Consequently, native reserves that were created to resettle the landless African farmers were located ‘haphazardly in infertile, low-rainfall potential areas and which subsequently became communal areas’ (Mafa et al. 2015:38; Potts 2010:79). Unfortunately, African farmers being the custodians of this land were never consulted in the identification and distribution of their land. As a result, hundreds of thousands of African farmers lost their land and settled in native reserves, which ‘generally had poorer-quality land’ and isolated from the country’s economic hubs (Kramer 1997; Mafa et al. 2015; World Bank 2019b). Studies (Kramer 1997; Mafa et al. 2015; Palmer 1977) show that these colonial policies were meant to suppress production in peasant farming areas, as most European farmers failed to fully utilise all the land they possessed from African farmers. As highlighted by Palmer (1977:242), 14 million acres of land acquired by the Europeans from African farmers remained unused and unoccupied by 1925. Similarly, the BSAC introduced stringent policies such as the Hut Tax to force African farmers to work in white-owned farms, mines and factories (eds. Crush & Tevera 2010:63, 64; Mafa et al. 2015:38, 51). In addition, the Europeans charged hut taxes, used violence and undertook kidnappings (Mafa et al. 2015:40, 50). This clearly shows the intentions of the Europeans that were meant to destroy peasant agriculture and promote their economic interest.

The high population growth rates and scarcity of farming land in native reserves led to deteriorating ecological conditions in these areas. In response, the British Government introduced the Native Husband Act (1951) to curb the environmental and ecological conditions happening in those farming areas. This policy introduced new land management and conservation measures such as the provision of land ownership titles, the introduction of farming and livestock permits and put caps on livestock numbers (Mafa et al. 2015:45), among others. As no consultations were done with the African community, the Native Husband Act was deemed to fail from the start as it did not address the root causes (population growth versus land scarcity) for ecological and environmental degradation in these areas. Furthermore, before the coming of the Europeans, African farmers were master farmers on their own. In doing this, African farmers applied their indigenous knowledge systems (IKS) in growing and preserving their crops for future use (Palmer 1977:225). According to Palmer (1977) and Kramer (1997), Africans build underground granary facilities to preserve their food from the previous year’s harvest and practiced sustainable farming methods to sustainably recharge their lands’ nutrients after long periods of extensive use. The Europeans failed to build on these sustainable farming systems that were used by generations of African farmers; as a result, they came with their Eurocentric farming systems that were not compatible with the local conditions. After years of suffering under this European colonial system, the Africans declared the first *Chimurenga War* (Liberation War) in 1896–1897 and that led to the death of high-profile Shona spiritual leaders that include Mbuya Nehanda and Sekuru Kaguvi (Mafa et al. 2015:41).

### Contemporary land policies and livelihoods in communal areas

Zimbabwe’s contemporary land development policies that came after independence (1980) also created several challenges for rural livelihoods. These contemporary development policies include the Land Reform and Resettlement Programmes (1980–2000), Economic Structural Adjustment Programme (ESAP) (1994) and Operation *Murambatsvina* [clear filth and/or restore order] (2005) led to serious suffering among communal farmers. The ESAP was a neo-liberal policy that was introduced by the Bretton Woods Institutions (IMF and World Bank) in Zimbabwe in the 1990s to reduce government expenditure. Operation Murambatsvina was a politically motivated government policy. The policy targeted urban populations by restoring sanity and decongesting the country’s urban areas. During Zimbabwe’s early years of independence, the new government introduced an urban-based economic development model that created economic hubs in urban centres and attracted labour from the marginalised areas of the country (Potts 2010:80). These policies including the early land reform and resettlement programmes were meant to promote economic development through employment creation and equal distribution of land resources (Logan & Tevera 2001:103; Sibanda & Makwata 2017:4, 7). All this changes the country’s demographic patterns as people flocked to urban areas and the new farming areas that were created by the government. According to Nyambara (2001), this development saw new farming areas such as Gokwe opening and attracting small-scale cotton farmers across the country. Furthermore, the government established institutions that include the Grain Marketing Board (GMB) and Cotton Marketing Board (CMB) that supported agricultural production and promoted food security in communal areas (Food and Agricultural Organization [FAO] 2003; Nyambara 2001; Rukuni et al. 2006). Similarly, the new government improved African farmers’ access to agricultural extension services and financing and this helped to improve the socio-economic conditions in communal areas (FAO 2003; Nyambara 2001; Rukuni et al. 2006). Several infrastructure development projects such as road construction were implemented, and this improved farmers’ accessibility to markets (Nyambara 2001).

Despite all these development, environmental degradation challenges continued to exist in communal areas. According to Logan and Moseley (2002:3), African farmers prioritised their economic needs ahead of environmental issues. As a result, scarcity of arable land remained a big challenge for most African farmers, and most people faced with these challenges resorted to farming in unauthorised areas such as grazing land, riverbanks and mountains (Logan & Moseley 2002:10; Nyambara 2001:258). Unfortunately, the various land programmes that were introduced by the government after independence neither empowered African farmers nor did they address the socioeconomic and environmental challenges in communal areas. This is despite reports by Moyo and Chambati (eds. 2013) indicating that approximately 13 million hectares of land were transferred from the hands of the Europeans to Africans by 2009. Instead of helping the economically marginalised communal people, these programmes transferred land usage rights from the Europeans to the African political cronies. According to Mafa et al. (2015), (Zembe et al. 2014) and Government of Zimbabwe (GOZ) (2015) many peasant farmers (not politically connected) were impoverished by these programmes because of lack of farming land and environmental degradation challenges caused by overgrazing and overexploitation of land and forest resources.

Compounding to these challenges in communal areas were the socioeconomic and environmental hardships brought about by ESAP. Economic Structural Adjustment Programme was introduced by the Bretton Woods Institutions as part of aid given to the Government of Zimbabwe in the early 1990s. Under ESAP, the Zimbabwe government was tasked to open its borders as part of encouraging free trade, undertake market reforms, devaluate the local currency and reduce subsidies (Logan & Tevera 2001:108), among other economic reforms. In the agriculture sector, ESAP reversed all the economic benefits enjoyed by small-scale farmers soon after independence. Thus, farmers were no longer entitled to agricultural input subsidies and agricultural financing (Matanda & Jeche 1998; Potts 2010; Rukuni et al. 2006). The prices of food and agricultural prices went up beyond the means of many farmers leading to increased livelihood challenges in communal areas (Potts 2010). Furthermore, small-scale farmers were now required to access their agricultural financing from banks, and this poses several challenges for those farmers who lacked collateral security (Rukuni et al. 2006). Farmers also suffered indirectly from the high exodus of Agricultural Extension Officers who fled to neighbouring countries because of the worsening economic conditions in the country.

### Climate change and food production

The severe climatic conditions caused by droughts, floods and storms being experienced in Zimbabwe have been increasingly impacted production in small-scale farming areas of the country. According to Brown et al (2012), IPCC (2014), Brazier (2015) and GOZ (2015), the turn of the 21st century has witnessed Zimbabwe experiencing an increase in severe climatic conditions. Studies by the GOZ (2017b) show that since 1950, Zimbabwe has been experiencing high episodes of mild, severe and extreme droughts. According to GOZ (2015), the country’s rainfall patterns have declined while annual surface temperatures have increased by 0.4°C since 1900. Climate projections indicate that the country will continue to experience warmer days by 2100 (GOZ 2015). There is no doubt that these climatic variations continue to pose serious livelihood challenges for communal farmers. Reports by Brown et al. (2012), GOZ (2015) and Brazier (2017) show that the drought conditions in the northern and southern parts of Zimbabwe have reduced the amount of water available for crops and livestock production. Historically, these are the farming regions located as AER IV and V with poor soils and rainfall patterns, which are below 400 mm per year that was allocated to African farmers during the colonial era (FAO 2006; GOZ 2016; Southern African Development Community [SADC] 2015).

The agricultural production challenges in these poor areas continue to be exacerbated by the lack of irrigation facilities that are needed to support peasantry agriculture, especially during this period of climate change (United Nations General Assembly [UNGA] 2020:9). Droughts have decreased communal farmers’ desire to be productive and this has been one of the reasons why the country has been experiencing a decrease in areas reserved for cereal production (SADC 2013, 2016). The same farming regions continue to be exposed to tropical cyclones that have resulted in flooding, waterlogging of crops and the leaching of soil mineral nutrients (GOZ 2015; Nangombe 2014; SADC 2017a, 2017b). As a result, poverty is rife in Zimbabwe’s communal areas occupied by small-scale farmers. Reports by the Famine Early Warning Systems Network (FEWS NET) indicate that over 5 million farmers were severely impacted by droughts during the 2018–19 seasons (New Zimbabwe 2019, March 08). According to the Global Hunger Index, the dire food insecurity challenges brought about by the droughts have seen the country’s hunger index increasing from 16.5 to 30.8 between 2014 and 2015 (GOZ 2017a). The food insecurity challenges that have besieged small-scale farmers are also attributed to the lack of livelihood diversification programmes in rural areas and the collapse of wage employment in urban areas, which normally provide safety nets for most rural people during periods of climatic stress.

## Theoretical framework

There is no doubt that poor climate adaptation in communal areas is a result of compounding multi-vulnerability factors caused by the country’s colonial history, contemporary development policies and extreme climatic conditions. Therefore, the authors contend that there is a need to first understand the complex development trajectory undertaken by Zimbabwe that makes climate change adaptation difficult in communal farming areas. This process includes examining the country’s complex political and historical context that created and led to livelihood challenges in these communal farming areas. Firstly, there is a need to understand the impact of the country’s contemporary development policies that followed independence in making climate change adaptation difficult in these poor areas. Lastly, it needs to be acknowledged that the severe climatic conditions caused by droughts and cyclones have not made life easier for communal farmers. Given this, both climatic and non-climatic conditions are working together to create livelihood challenges for farmers in communal areas. In order to understand this relationship, this study adopts a contextual and historical approach (Morrissey 2012). Through using a contextual and historical approach, this study is better positioned to understand the impact of historical and contemporary development decisions that have made climate change adaptation difficult in these poor areas. The CHA goes further to establish:

[*T*]he reasons why structures look the way they do, and begin to think why people will move, what impacts of that movement might be and, what measure might be taken to best secure human well-being. (Morrissey 2012:46)

The authors argue for a climate change policy that takes into consideration both climatic and non-climatic factors. This includes an understanding of the country’s historical and contemporary development strategies brought about by colonialism and international development strategies for effective climate change adaptation in communal areas.

## Methodology

### Research areas

**FIGURE 2:**
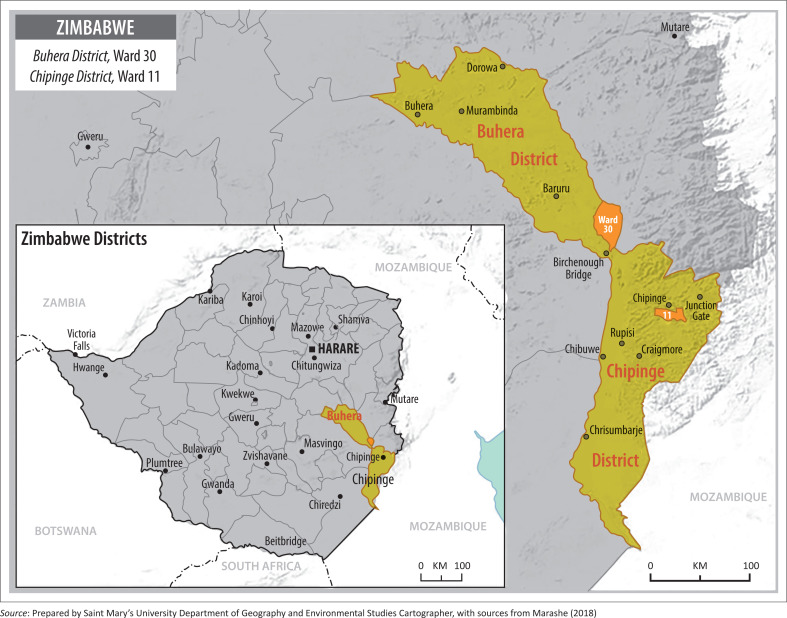
Map of Buhera and Chipinge study areas.

This study was conducted in Zimbabwe’s Buhera Rural District (Ward 30) and Chipinge Rural District (Ward 11). These districts are found in Manicaland Province (see Figure 2). According to Zimbabwe National Statistics Agency (Zimstat) (2017), Manicaland Province is located in the eastern highlands, has a total area of 36 456 km^2^ of land and 1.8 million people. This province encompasses all the AERs (I–V) of Zimbabwe and has several perennial rivers that make agriculture viable in most of its regions (Chingarande et al. 2020). Buhera rural district is in the south-western regions of Manicaland Province. There are 33 wards, 256 462 people and 57 000 households with an average household size of 4.3 persons per household in Buhera (Zimstat 2012). Approximately, two-thirds of the land in Buhera is occupied by communal farmers and falls under AERs IV and V (Oxfam 2015). These are low veldt farming areas that are limited to subsistence farming. Farmers in these areas specialise in growing small grains (millet and sorghum) and livestock production for their consumption (Chingarande et al. 2020; FAO 2020; Oxfam 2015; Zamchiya 2011). On the other hand, Chipinge rural district is found in the south-eastern regions of Manicaland Province. It has 30 wards, occupied by over 300 000 people, and 66 403 households with an average household size of 4.5 persons (Zimstat 2012). According to FAO (2006) and Chingarande et al. (2020), this district consists of large tracts of land in high veldt areas (AERs I–II) that is primarily used for diversified large-scale commercial farming. This farming area thrives well for dairy and beef production, including a variety of food crops (Chingarande et al. 2020; FAO 2006; Zamchiya 2011).

These two districts represent two distinct regions in terms of their historical development context and agroecological conditions. Chipinge is located in former white commercial farming regions, and it boosts fertile lands, rich biodiversity and favourable climatic conditions that average approximately 1000 mm of annual rainfall (FAO 2006). The favourable agro-ecological and climatic conditions found in Chipinge have enabled farmers in this region to venture into growing high-value cash crops that are mainly meant for exports (Chingarande et al. 2020; FAO 2006). Over the years, white commercial farmers used to benefit from massive government investments to support their production. This support came in different forms ranging from infrastructural development, access to lines of credit and agricultural markets (Kramer 1997; Mafa et al. 2015; Potts 2010). On the other hand, lands in Buhera are poor, continuously subject farmers to unreliable rainfall patterns of below 450 mm per year and expose farmers to growing low-value crops (Chingarande et al. 2020; FAO 2006). Historically, these were Tribal Trust Lands that accommodated African farmers who lost their lands to the Europeans during the colonial era. Despite all the financial and technical support that was offered to white farmers, no meaningful development and investments were done in African farming areas (Kramer 1997; Mafa et al. 2015; Potts 2010).

### Data collection

Data collection involved the use of individual household interviews, with the use of a snowball sampling method (Buhera = 34, Chipinge = 14), focus group discussions (7), key informant interviews (29) and direct observation in the field. In Chipinge, households were selected from Zimbabwe’s AERs 4 and 5. Given that these were large-scale farming areas previously occupied by white commercial farmers, the households were far from each other, which explains the lower numbers of interviewees in this area. Males dominated most individual household interviews because of the patriarchal nature of Zimbabwe’s rural economies. Focus group discussions were utilised to get women to participate during the field study. Households were selected based on their knowledge and lived experiences in the areas under study and snowball sampling was used for expert interviews. In Buhera district, households were required to meet the following criteria (1) lived in that area for more than 10 years and practicing communal farming, (2) have experienced more than two climatic disasters during the last 15 years or so and (3) have experience working as casual labour or engaged in trade in AERs I and II. In the Chipinge district, households who migrated from either Buhera or AERs IV and V of the country were selected. These households either moved (1) permanently and now own land, (2) temporarily and working as casual labourers, or (3) are engaged in short-term trade in the Chipinge district. Participants for key informant interviews were selected based on their experiences working with communities in these farming regions. All Individual household interviews and key expert interviews were administered using semi-structured interviews with open-ended questions. Those interviewed included heads of households, local leaders, officials from government agencies and members of non-governmental organisations.

### Data analysis

This process linked field data with the study’s research questions. This involved breaking ‘the text down into smallest units and reorganising these units into relatable stories’ (Yi 2018). The authors spent a considerable amount of time transcribing the interviews captured during fieldwork. This study employed an inductive approach to qualitative data coding. The inductive approach to qualitative data coding uses a bottom-up approach to qualitative data coding, thus it allowed them to derive their codes from the field data (Asher Consult 2014; Blackstone 2014; Yi 2018). All the major themes were identified and manually coded using a pen and paper; hence, no software was used in the analysis of the qualitative data.

### Ethical considerations

This article followed all ethical standards of research. All terms and conditions guiding this research were approved by the Saint Mary’s University Research Ethics Board (SMU REB registration number: 19-052).

To maintain the confidentiality of the informants, the information used in this publication cannot reveal the identity of the individuals. This study also got approval from the Government of Zimbabwe to do fieldwork in the Buhera and Chipinge farming areas.

## Results and discussions

### The nexus between colonial land policies and livelihood challenges in communal areas

Through discussions with communal farmers, it was learned that the impact of colonial land policies in Zimbabwe are still affecting their food insecurities. It was observed that colonial policies continue to play a critical role in explaining the suffering that most African farmers went through during and after the colonial rule in Zimbabwe. The *Land Apportionment Act* of 1930 disenfranchised land according to racial land. Nothing much was done by the new Zimbabwe government to change to improve the economic well-being of farmers who were affected by this policy. In fact, the contemporary land reform programme mimicked the old colonial policy, and it is only that land in new Zimbabwe was redistributed according to class and political connections. For those without political connections especially communal farmers suffered from these land ownership injustices as they continued to be exposed to poor lands. Sadly, despite the country obtaining its independence 40 years ago, poverty is still rife in communal areas. Overcrowding and land degradation continue to persist resulting in poor agricultural production in these areas (Logan & Moseley 2002; Mafa et al. 2015). One government official said:

‘[*C*]olonial decisions led to serious overcrowding in communal areas, and these decisions are still having an impact on people’s livelihoods today, including increasing their desires to move. Areas in Buhera and other marginal areas have been overpopulated and overstocked for long and this has led to dire economic and environmental consequences stemming from high land degradation, erosion and siltation of water bodies that continue to reduce the irrigation capacity of rivers.’

Similar sentiments were also shared by an international NGO official who said:

‘[*T*]he land-use policies have not changed since the colonial period as more and more African farmers continue to be trapped in marginal regions including flood plains of the Zambezi Valley and other drier regions.‘

According to the Zimbabwe Census, the county’s population grew from 7.5 million to approximately 13.1 million people between 1982 and 2012 (GOZ 2015; Zimstat 2012, 2013). The majority of this population lives in rural areas and no meaningful development was done by the government to address the socio-economic and environmental challenges brought about by these demographic changes in communal areas (GOZ 2015; Zimstat 2013). There is no doubt that the country’s demographic changes continue to pose serious livelihood challenges for most rural dwellers. Some serious environmental and ecological challenges brought about by land scarcity and high populations in communal areas were also observed. Examples of what was observed included many individuals now practicing stream bank cultivation along the Save River, some resorting to grazing land and mountain areas. Deforestation was also high in these areas as people cleared forest for farming land and for firewood. Gullies were also developing in Buhera because of deforestation. Similar issues were raised during one of the interviews with a Buhera senior district official during fieldwork who said:

‘[*W*]e are having serious environmental issues in this district (Buhera) because of overcrowding. People are overcrowded and lack suitable farming land and as a result, they are plowing down the slopes and not constructing contour ridges. These unsustainable farming practices have exposed most lands to soil erosion and degradation. There also have cases of people with no farming land at all, unfortunately, these are the same people farming along the Save Riverbanks or occupying land in mountainous areas which further exposes them to extreme climatic events and hunger.’ (Buhera, Senior District Official)

Also, an international NGO official said:

‘The high population growth rates versus the limited land available for farmers in these poor regions have seen many people settling in mountainous areas in Manicaland with limited land for farming and livestock production, thus people continue to be trapped and vulnerable.’

Through our focus group discussion with the youths in Buhera, it was learned that streambank cultivation is not one of their desired livelihood options given the environmental degradation challenges associated with it, but the youths have no choice as it is now a matter of survival given the lack of fertile land on the mainland.

Unlike Buhera, the commercial farming lands in Chipinge that benefited from European occupancy (now occupied by A1 and A2^3^ African farmers after the Fast Track Land Reform Program (FTLRP) have large tracts of land and a well-developed infrastructure that includes good road networks, functional market systems and reliable water sources and irrigation systems. As highlighted by one government official from Harare:

‘Chipinge district is in areas with better climatic conditions [more favourable conditions for agriculture] and many land beneficiaries benefited through the Land Reform Programme… there is no doubt that most of these farmers are from marginal regions and they chose Chipinge because of its good soils and rains, a well-developed infrastructure including abundant pastures for their cattle. It is known that these people from marginalised regions are mainly looking for areas where they can utilise rains for their agriculture which is not mechanised.’

Similarly, one migrant farmer now in Chipinge said:

‘[*T*]he reasons for my departure from Sabi Valley are scarcity of land because of overcrowding, poor agricultural infrastructure that we inherited from our colonisers and the persistent droughts that caused acute food shortages. The land reform programme presented me with an opportunity to come to Chipinge where I am living like a king as I now realise good harvest and have sustainable incomes to send my children to school and to live the life I need.’ (a 50-year-old, male)

One international NGO Official also weighed in on this discussion and said:

‘[*T*]he current human mobility happening in communal areas have a historical component in it. These people were placed in marginal lands with poor soils that are now exhausted and need massive fertiliser investment which I see not feasible because of their poor economic situation and economic imbalances. So why should these people continue to suffer when they used to have a good place to stay in their motherland? I tell you that is the major reason why people prefer to go back to their traditional lands before colonisation, just to boost their socioeconomic statuses.’

According to the Zimbabwe National Statistics Agency, approximately 482 621 and 50 715 households are resettled under the A1 and A2 farming models, respectively, across the country (Zimstat 2015a, 2015b). According to the 2014 and 2019 Labour Force Surveys, approximately 126 000 people have moved across the country’s districts in search of better agricultural land (Zimstat 2015c:226, 2020:215). It is undisputable that the high population movements into Chipinge’s AERs I and II are mostly performed by people coming from the drought prone AERs IV and V of the country. Colonial land and contemporary agricultural policies did little justice in supporting or investing in agricultural systems owned by African farmers. These issues came out during our discussion with a peasant farmer from Buhera. He said:

‘Our suffering here is well connected to the land policies brought about by Ian Smith (Former Prime Minister of Rhodesia/Zimbabwe) that disenfranchised African Farmers from the good lands… we had high hopes that President Mugabe was going to help us acquire good lands but nothing changed…we are still farming in these marginal lands and now poor soils are exhausted and there is nobody to assist us with fertilisers, which is the major reason we are having recurrent food shortages here…now with these droughts, I do not know what to do… sometimes I contemplate tracing back my roots to Chipinge but as you can see, I am too old for that now.’ (a 80-year-old Buhera, male)

Similar sentiments were shared by one female farmer in Buhera who said:

‘I inherited this piece of land from my grandfather, and he also got it from his grandfather who has been using it also for several years. Now the land is barren as a result of being overused and even if I apply fertiliser or it rains nothing much is changing… the government needs to avail new lands to us sooner or we will all end up deserting this place.’ (a 30-year-old Buhera, female)

We also need to acknowledge that over the years Zimbabwe has witnessed high population movements across the country’s AERs because of severe natural hazards caused by cyclones. For example, the recent Cyclone Idai (2019) saw many people moving from AERs I and II to AERs IV and V. Apart from killing thousands of people and destroying agricultural fields, Cyclone Idai also displaced more than 2000 people (Sibanda n.d.), who later settled in some of the country’s marginal regions. These issues were observeds in our interview with a Buhera senior district official, who said:

‘[*M*]any people are moving from Chimanimani to Buhera because of the cyclone Idai disaster that fell into them and destroyed their homes and agricultural fields. They found it much safer here in Buhera, and this is because of limited natural hazards of that nature in this district.’

From these discussions with communal farmers, it can be concluded that living in these marginal areas is no longer a viable option for them because of the deplorable living conditions that were created by the colonial policies and inherited by their ancestors. It is undeniable that the colonial systems drove African farmers from their prime agricultural lands, thus exposing them to serious production challenges. Contemporary development policies did little justice for these farmers as they continue to suffer from underdevelopment and severely impacting their drive to achieve their food security.

### Zimbabwe’s macroeconomic challenges and climate adaptation

Lack of meaningful development policies and the country’s macroeconomic challenges have exacerbated poverty in communal areas. As highlighted by Maganga and Conrad Suso (2022) and the United Nations Development Program (UNDP) (2017), the lack of job and income diversity opportunities, hyperinflation and shortage of agricultural inputs coupled with the extreme climatic conditions have exacerbated food insecurity in communal areas. This was highlighted by many interviewees:

‘We are living in desperate times here, firstly the rains are not coming and you have no food to give the children, secondly the food prices are going up every day, and you sometimes wonder if this life is worth living for.’ (a 40-year-old Buhera, female)

These poor economic and environmental conditions have made life difficult in communal areas thereby prompting people to move to areas that guarantee them better economic opportunities and food security (FAO 2016; Maganga & Conrad Suso 2022; World Bank 2019a):

‘The ever-rising prices of and basic food commodities such as maize meal, sugar and cooking oil have made life impossible for us widows, we have resigned to ourselves to our fate as we have no one to take care of us.’ (a 40-year-old Buhera, female)

These challenges were highlighted by a Buhera communal farmer during one of theinterviews. She said:

‘Firstly, the macroeconomic challenges have contributed to hunger in this area (Buhera) and have seen most young people leave their home areas to look for greener pastures somewhere else. Secondly, the price economy is forcing people to leave depressed farm incomes in marginal areas into urban areas, as key crops are not fetching good prices.’ (a 40-year-old Buhera, female)

Similar sentiments were also shared by one government official from Harare, who said:

‘[*T*]he majority of people are leaving the rural areas and going to towns (not because of bad climate) but because of the rural economy, which is not functioning well… Zimbabwe is a cash economy, so without cash, people cannot sell or buy anything.’

Furthermore, these climatic (both droughts and flooding) and macroeconomic challenges have resulted in high cases of extreme poverty rates rendering most government and donor-led social programmes useless in marginalised areas. As highlighted by Mtetwa and Muchacha (2013:19), the high poverty cases in communal areas have made most social protection programmes ineffective, as they continuously fail to address the socio-economic challenges facing communal people. These challenges were observed during fieldwork with these issues brought forward by two government officials working for the Social Welfare Department in Buhera and Chipinge who said: ‘… but the numbers have since gone up [of food-insecure households] because of climatic and weather patterns affecting farm production in the region’ (Buhera, Government Official, 6) and ‘the number of food-insecure households in the region is increasing every year, as there is more request for food aid in most areas’ (Chipinge, Government Official). The information collected during field studies showed that the country’s social protection programmes suffer from low unpredictability, poor coverage and transparency. As highlighted by Mtetwa and Muchacha (2013) and World Bank (2016), these programmes that include cash transfers and food aid programmes are highly infiltrated by politicians and they only serve a few people; therefore, they are not making any significant progress in addressing the looming food security challenges in communal areas. According to one Buhera farmer:

‘As far as I know, we have the social welfare programmes that are giving people grain and cash but getting into these programmes is hard as they focus on elderly and widows although everybody has been affected by droughts in this region.’ (a 40-year-old Buhera, male)

Furthermore, the macroeconomic challenges facing the country have eroded the value of aid given to vulnerable communities. For example, over the years, the Government and NGO-led Harmonised Social Cash Transfer (HSCT) programme continuously suffer from hyperinflation and the aid given no longer meets the needs of the people (World Bank 2016). One HSCT beneficiary had this to say:

‘This money is valueless given the hyperinflation and daily food prices we are enduring every day …the money I am receiving is too little to meet my daily food requirements together with my family… you cannot even buy a bucket of maize and a bottle of cooking oil with that money.’ (a 45-year-old Buhera, female)

There is no doubt that these worsening socioeconomic conditions have lowered the living standards in both urban and rural areas of the country. Consequently, these challenges have seen Zimbabwe recording a ‘Low’ Human Development Index (HDI) of 0.51 making it one of the poorest countries in the world (Chereni & Bongo 2018:18).

From this discussion, it is clear that the prevailing socio-economic and environmental challenges found in communal areas have made climate change adaptation difficult in communal areas. These challenges that have been exacerbated by poor social protection programmes, hyperinflation, forex shortages, an exodus of Agricultural Extension Officers (AREX) and climate change have made it almost impossible for most rural households to sustain their livelihoods.

## Conclusion and recommendations

The authors ascertain that the current food insecurities and poor climate change adaptation happening in Zimbabwe’s communal areas can best be understood using a colonial and political historical lens. It is undisputed that the country’s colonial legacy that led to the establishment of communal areas in marginal regions of the country and inherited by the new black-led government in 1980 is contributing to the suffering of African farmers located in these poor regions. Poverty is still rife and farmers are continuously suffering from poor production, thus increasing people’s vulnerability to climate change. In this context, an understanding of climate change vulnerability in Zimbabwe’s communal areas using a colonial and historical development perspective enabled this study to establish the nexus between colonial and contemporary developmental policies, and subsequently the underdevelopment of peasant agriculture, livelihood stresses and adaptation challenges faced by communal farmers. No meaningful development has happened in these poor regions since independence (40 years ago) as the majority of farmers still farm on barren lands, rely on rain-fed agricultural systems and lack income diversification opportunities to support their livelihoods. Furthermore, post-independence economic development policies introduced by the government such as the land reform programmes have failed to empower peasant farmers, while the globally induced economic development policies (ESAP) and economic sanctions have exacerbated the socioeconomic, environmental challenges in communal areas. The interplay of these factors has made climate adaptation complex in communal areas.

Given this, Zimbabwe’s policymakers need to recognise the country’s colonial and historical legacy that has led to poverty and other livelihood challenges in communal areas. Through understanding this, policymakers are better informed about the structural issues making climate change adaptation difficult in these regions. By understanding this, policymakers could intervene more efficiently by tailoring their strategies to suit the adaptation challenges being faced by communal farmers. The authors contend that the failure by policymakers to understand these complex political historical–structural issues, climate change adaptation in communal areas will remain a mirage. Furthermore, Zimbabwe should refrain from the ‘one size fits all’ policies being promoted by the international community that does not suit its political and economic context. Instead, Zimbabwe should align its climate change adaptation policies against the background of its colonial history that stems from its long periods of suffering under the British Government. This requires the government to be honest with itself by removing all the biases and blind spots and revisiting the land ownership issues affecting agricultural production in the country. For this to work, the meaningful involvement of marginalised farmers while framing the implementation of these land redistribution programmes to address production challenges in communal areas is recommended.

## Footnotes

1 ‘The British South Africa Company (BSAC) was a mercantile company incorporated on 29 October 1889 by a royal charter given by Lord Salisbury, the British prime minister, to Cecil Rhodes. The company was modelled on the East India Company and was expected to annex and then administer territory in south-central Africa, to act as a police force and develop settlements for European settlers. The charter was initially granted for 25 years and was extended for another 10 years in 1915’ (Boddy-Evans 2017).

2 There are five AERs in Zimbabwe classified according to their soil quality, rainfall and temperature conditions. AER I and II are intensive farming regions with annual rainfalls of >1000 mm and 750–1000 mm, respectively; AER III is a semi-intensive farming region with an annual rainfall of 650–750 mm; AER IV is a semi extensive farming region with annual rainfall of 450–650 mm and AER V is an extensive farming region with annual rainfall of ≤ 450 mm (Food and Agricultural Organization [FAO] 2006).

3 These are government-designated programmes that came during the FTLRP. Under the A1 farming model, individual families own at least 6 ha of land. These families have village homesteads with farming areas that are located in designated areas, including common grazing lands for livestock (Zimstat 2019:18). On the other hand, A2 farmers own large tracts of land (depending on their AERs) used for both crop and livestock production (Zimstat 2019:18). A2 farming models are similar to commercial farming models, as farmers are provided with the offer letter and a 99-year lease from the government (Zimstat 2019:18).
